# Supplementary material for: Ligand dependent interaction between PC-TP and PPARδ mitigates diet-induced hepatic steatosis in male mice
Source: Nat Commun. 2023 May 12;14:2748. doi: 10.1038/s41467-023-38010-w (PMC10182070; doi:10.1038/s41467-023-38010-w)
Supplement: Supplementary file 1 — Supplementary Information [file 41467_2023_38010_MOESM1_ESM.pdf]

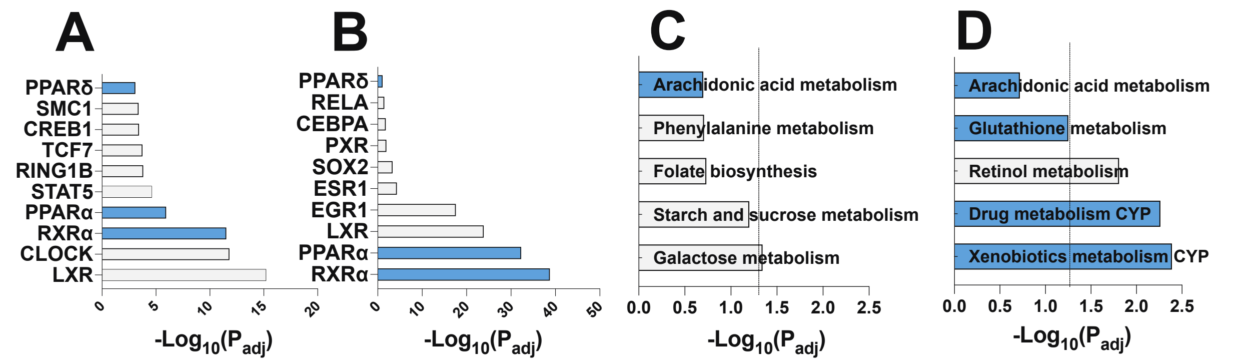

Supplemental Figure 1. RNAseq comparing *Pctp*$^{-/-}$ and WT chow and MCD fed mice. RNA from chow and MCD fed WT (n = 3) and *Pctp* $^{-/-}$ (n = 3) mouse livers was used for RNAseq analysis. Statistical significance was determined using the Wald test followed by the Benjamini Hochberg correction. Significantly altered analytes were then analyzed via Enrichr for pathway analysis. **A & B**) Enrichr analysis of DEGs from chow fed and MCD fed mice respectively, mice were compared to know transcription factor CHIP-seq databases (CHEA).**C & D)** Enrichment analysis of altered metabolic pathways (KEGG) determined by cross referencing the statistically significantly altered genes for chow and MCD fed mice respectively.

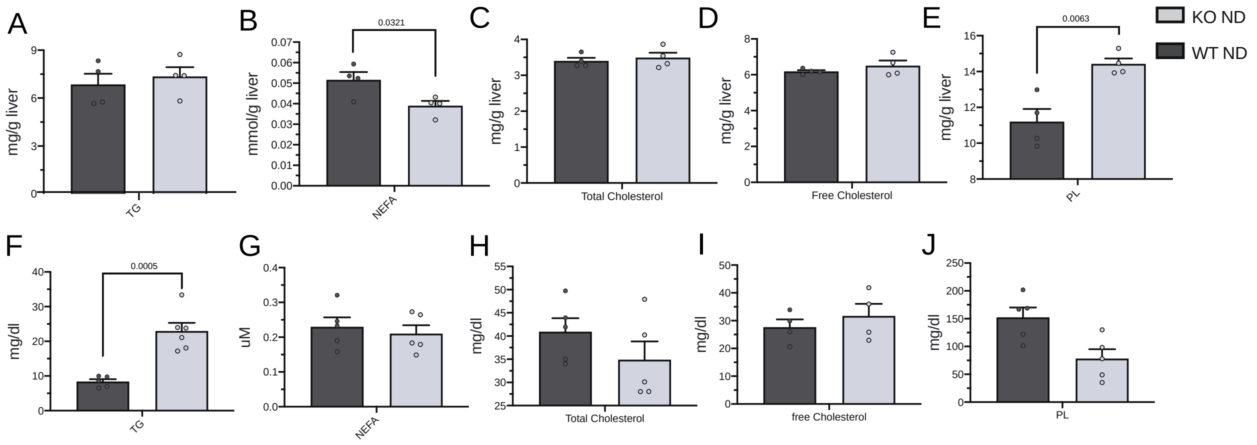

Supplemental Figure 2. Characterization of liver and plasma the L-*Pctp*$^{-/-}$ mouse fed ND. A-E) Quantification of liver composition of triglycerides (TG), free fatty acids (FFA), phospholipids (PL), as well as total and free cholesterol for L-*Pctp*$^{-/-}$ and WT mice (n=5, T-test, SEM). **F-J)** Quantification of Plasma composition of triglycerides (TG) (n_{wt}=5 n_{ko}=6), Non-esterified fatty acids (NEFA) (n=5), as well as total cholesterol (n=5) and free cholesterol (n=4) and phospholipid (n=5) for L-*Pctp*$^{-/-}$ and WT mice (Two-tailed T-test, SEM).

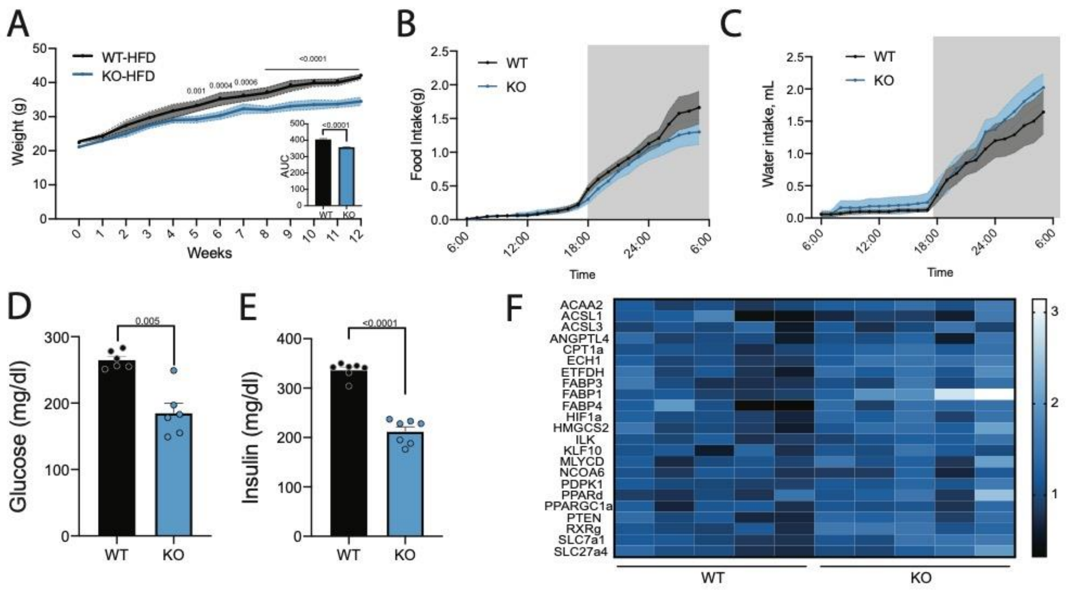

Supplemental Figure 3. Additional characterization of the L-*Pctp*$^{-/-}$ mouse fed HFD. A) Growth curve of L-*Pctp*$^{-/-}$ (KO) and WT mice on HFD throughout the period of diet intervention, with quantification of area under the curve provided in the insert (n=15, Two-tailed T-Test, SEM). **B & C)** Food intake and water intake of HFD cohort measured over one day show no significant difference in nutrient consumption to explain changes in weight (n=6, Two-tailed T-Test, SEM). **D & E)** Measure fasting glucose and insulin in KO and WT mice on HFD (n=6, Two-tailed T-test, SEM). **F)** qRT-PCR of cDNA generated from the livers of WT and KD mice fed HFD shows significant alteration in several genes known to be regulated by PPARs. (n=5, Two-way ANOVA, SEM).

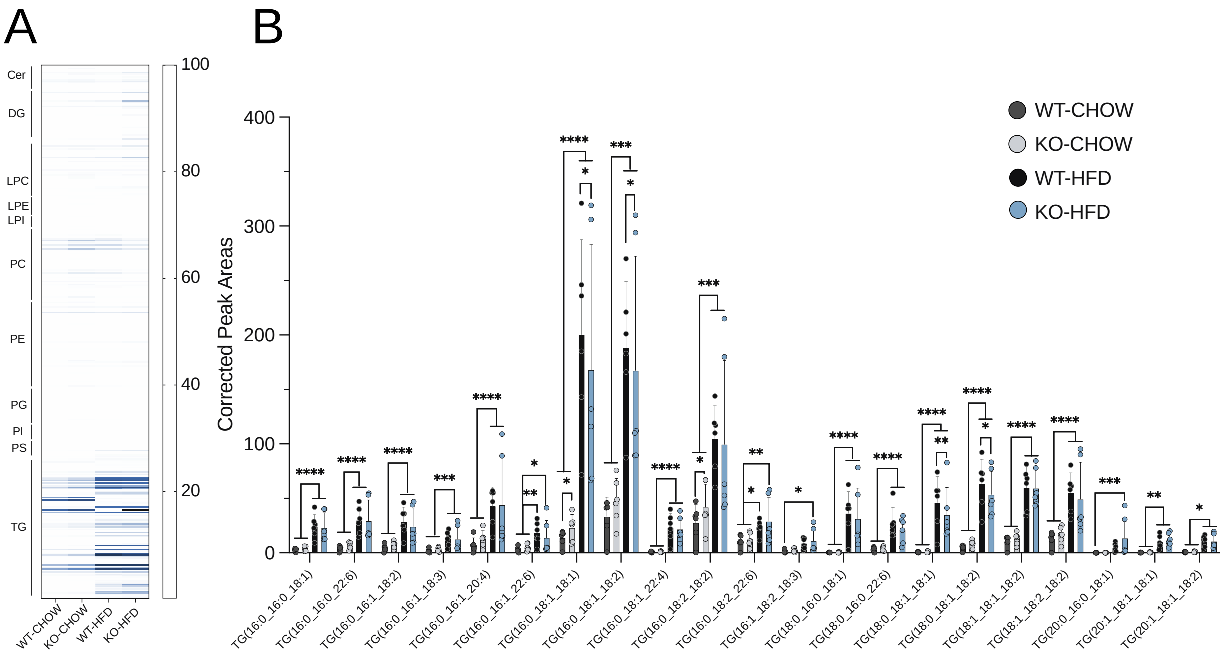

Supplemental Figure 4. Analysis of liver lipid composition by untargeted lipidomics. A) Heatmap of high confidence calls isolated from our untargeted lipidomics analysis of whole livers, grouped by lipid class (n=6). **B)** Significantly alter lipid species as determined from our untargeted analysis shows only TGs as differentially regulated by diet and/or deletion of PC-TP (n=6, Two-way ANOVA, SEM).

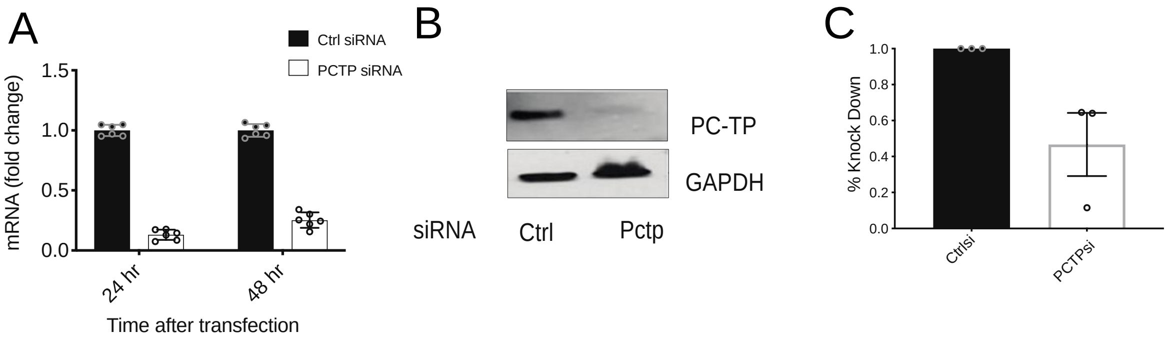

Supplemental Figure 5. Validation of siRNA mediated knockdown in Huh7
A) qPCR shows efficient knockdown of gene expression of PC-TP at 24 and 48 hours (n=6, SEM). **B)** Representative western blot further demonstrates loss of steady state protein levels. **C)** Quantification of intensities of western blot analysis of PC-TP KD (n=3, SEM)

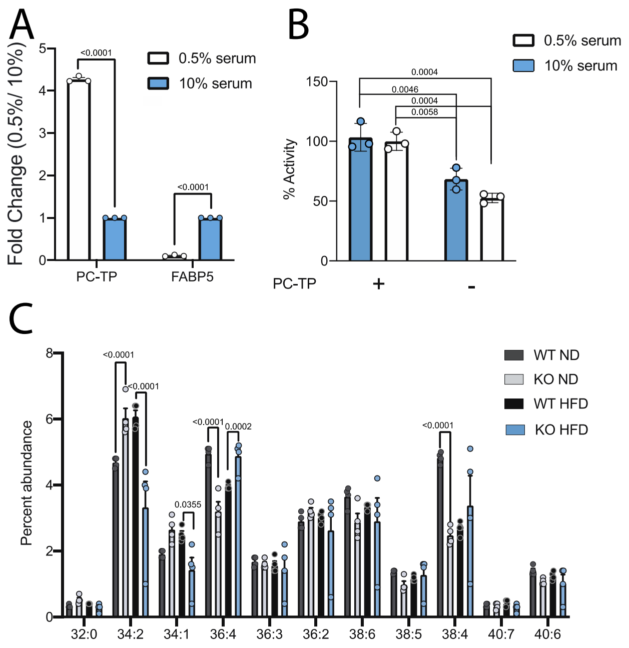

Supplemental Figure 6 Additional evidence of ligand mediated repressive complex. A) PC-TP-PPARδ or FABP5- PPARδ interaction detected in Huh7 cells was significantly altered by restricting serum (n=4, Two-tailed T-Test, SEM.) **B)** Luciferase reporter data performed in Huh7 cells confirms the PC-TP repression of PPARδ activity with a slight decrease in PPARδ activity in serum starved Huh7 cells (n=3, Two-way Anova, SEM.). **C)** Lipidomic profiling of the PC species isolated from livers of *Pctp*$^{-/-}$ on normal diet (ND) or high fat diet (HFD) reveals changes in select PCs (n=4, Two-way ANOVA, SEM).

Supplementary Table 1. Data collection and refinement statistics.

	**PC-TP PC(36:4)**
**Wavelength**	1
**Resolution range**	33.76 - 2.185 (2.263 - 2.185)
**Space group**	I 4 2 2
**Unit cell**	133.172 133.172 83.271 90 90 90
**Total reflections**	5074487
**Unique reflections**	19663 (1906)
Multiplicity	
Completeness (%)	99.46 (98.08)
Mean I/sigma(I)	14.9
Wilson B-factor	61.42
R-merge	
R-meas	1.543

R-pim	0.326
CC1/2 in highest resolution shell	0.75
Reflections used in refinement	19605 (1888)
Reflections used for R-free	980 (93)
R-work	0.2322 (0.4949)
R-free	0.2433 (0.4791)
Number of non-hydrogen atoms	1767
macromolecules	1667
ligands	60
solvent	40
Protein residues	203
RMS(bonds)	0.012
RMS(angles)	1.58
Ramachandran favored (%)	94%
Ramachandran allowed (%)	6%
Ramachandran outliers (%)	0%
Rotamer outliers (%)	8%
Clashscore	7
Average B-factor	85.33
macromolecules	86.05
ligands	74.32
solvent	76.59
Number of TLS groups	1

Statistics for the highest-resolution shell are shown in parentheses.

Supplementary Table 2. Layout of custom RT2 microarray CLAM28936C

	1	2	3	4	5	6	7	8	9	10	11	12
A	Acaa2	Acsl1	Acsl3	Angptl4	Acaa2	Acsl1	Acsl3	Angptl4	Acaa2	Acsl1	Acsl3	Angptl4
B	Cpt1a	Ech1	Etfdh	Fabp3	Cpt1a	Ech1	Etfdh	Fabp3	Cpt1a	Ech1	Etfdh	Fabp3
C	Fabp1	Fabp4	Hif1a	Hmgcs2	Fabp1	Fabp4	Hif1a	Hmgcs2	Fabp1	Fabp4	Hif1a	Hmgcs2
D	Ilk	Klf10	Mlycd	Ncoa6	Ilk	Klf10	Mlycd	Ncoa6	Ilk	Klf10	Mlycd	Ncoa6
E	Pdpk1	Ppard	Ppargc1a	Pten	Pdpk1	Ppard	Ppargc1a	Pten	Pdpk1	Ppard	Ppargc1a	Pten
F	Rxrg	Slc7a1	Slc27a4	Scd1	Rxrg	Slc7a1	Slc27a4	Scd1	Rxrg	Slc7a1	Slc27a4	Scd1
G	Actb	B2m	Gapdh	Gusb	Actb	B2m	Gapdh	Gusb	Actb	B2m	Gapdh	Gusb
H	Hsp90ab1	GDC	RTC	PPC	Hsp90ab1	GDC	RTC	PPC	Hsp90ab1	GDC	RTC	PPC

Supplementary Table 3. Sequences of oligonucleotides used for qPCR are presented

Gene	Primer direction	Sequence (5’-3’)
**ADRP**	forward	TGTGAGATGGCAGAGAACGGT
**ADRP**	reverse	CTGCTCACGAGCTGCATCATC
**HMGCS2**	forward	GGAACCCATATGGAGAATGTGT
**HMGCS2**	reverse	ATCGCTGCCAGCTTGCTT
**PPARD**	forward	GTCACACAACGCTATCCGTTT
**PPARD**	reverse	AGGCATTGTAGATGTGCTTGG
**MCAD**	forward	TTCCAGAGAACTGTGGAGGTCTT
**MCAD**	reverse	TCAATAGCAGTCTGAACCCCTGT
**PEPCK**	forward	TGCATGAAAGGTCGCACCA
**PEPCK**	reverse	CACAGAATGGAGGCATTTGACA
**ANGPLT4**	forward	GATGGCTCAGTGGACTTCAACC
**ANGPLT4**	reverse	CCCGTGATGCTATGCACCTTC
**LPCAT3**	forward	TGGGCCGCACCATCAC

**LPCAT3**	reverse	AGTTGCCGGTGGCAGTGTA
**ACSL4**	forward	CCCTGAAGGATTTGAGATTCACA
**ACSL4**	reverse	CCTTAGGTCGGCCAGTAGAAC

Supplemental Table 4. Chromatographic parameters for data acquisition.

Time (min)	A: 60:40 ACN:H2O	B: 90:10 IPA:ACN
0	80	20
3.0	80	20
7.0	40	60
11	40	60
20	5	95
29	5	95
29.1	80	20
30	80	20

Supplemental Table 5. Instrumental parameters for data acquisition.

Parameter	Setting
Ion Source Type	H-ESI
Spray Voltage	Static
Positive Ion (V)	3500 V
Negative Ion (V)	3500 V
Sheath Gas (arb. units)	50
Aux Gas (arb. units)	10
Sweep Gas (arb. units)	1
Ion Transfer Tube Temp (°C)	300
Capillary Temp (°C)	275
S-Lens RF Level	40V
Data Acquisition Strategy	Full scan and data dependent acquisition
Full scan resolution (FWHM)	120,000
MS/MS resolution (FWHM)	30,000
HCD Fragmentation:	Normalized collision energy (NCE) of 25, 35, 45, 55 eV
	Stepped NCE of 10, 20, 50 eV

A

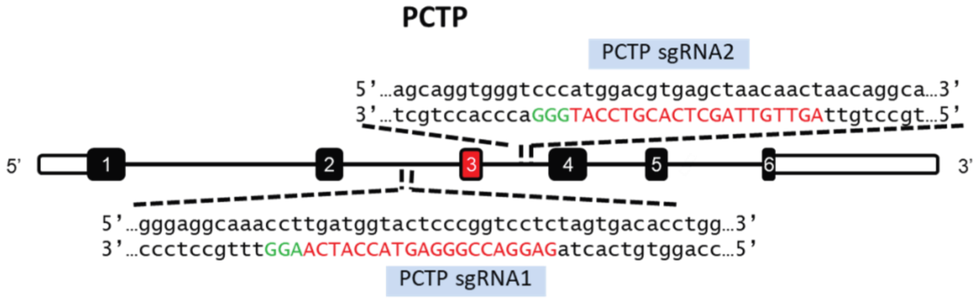

B

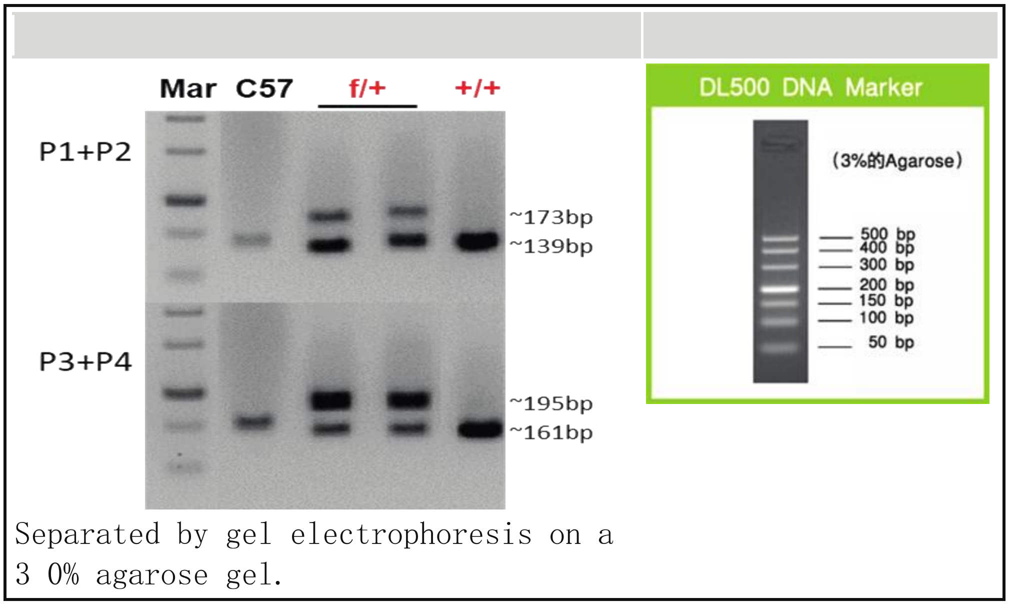

!

Primer	Expected Results:
P1+P2	Wild type = ~139 bp;<br>Heterozygote = ~139 bp & 173 3=;<br>Mutant = ~229 3=
P3+P4	Wild type = ~161 bp;<br>Heterozygote = ~161 bp & 195 3=;<br>Mutant = ~195 3=

Supplemental figure 7. Generation of the *Pctp*$^{flox/flox}$ mouse. A)The guide RNAs targeting specific loci in the genome will guide Cas9 enzyme to the locus and cause DNA double strain break (DSB).

Homologous recombination (HR) will enable the donor fragment (single-stranded DNA oligos) which contains loxP sites to integrate into the breaking locus. These mice possess loxP sites on either side of exon 3, which encodes the DNA binding domain. Mice that are homozygous for this allele are viable, fertile, normal in size and do not display any gross physical or behavioral abnormalities. When these mutant mice are bred to mice that express Cre recombinase, resulting offspring will have exon 3 deleted in the cre-expressing tissue(s).A pair of crispr/cas9 guideRNA constructs that were specifically targeted to PCTP intron 2 **B)** Validation of generation of the *Pctp*$^{flox/flox}$ mouse (n=2).
