# Supplementary material for: Ligand dependent interaction between PC-TP and PPARδ mitigates diet-induced hepatic steatosis in male mice
Source: Nat Commun. 2023 May 12;14:2748. doi: 10.1038/s41467-023-38010-w (PMC10182070; doi:10.1038/s41467-023-38010-w)
Supplement: Supplementary file 3 — Description of Additional Supplementary Files [file 41467_2023_38010_MOESM3_ESM.pdf]

Description of Additional Supplementary Files

Supplementary Data 1:

High confidence calls for analytes identified via our untargeted lipidomics analysis interrogating lipid levels in WT and KO mice fed HFD or CHOW (n=6)
