# Supplementary material for: Ligand dependent interaction between PC-TP and PPARδ mitigates diet-induced hepatic steatosis in male mice
Source: Nat Commun. 2023 May 12;14:2748. doi: 10.1038/s41467-023-38010-w (PMC10182070; doi:10.1038/s41467-023-38010-w)
Supplement: Supplementary file 5 — Reporting Summary [file 41467_2023_38010_MOESM5_ESM.pdf]

Corresponding author(s): Eric Ortlund
Last updated by author(s): Nov 30, 2022

Reporting Summary

Nature Portfolio wishes to improve the reproducibility of the work that we publish. This form provides structure for consistency and transparency in reporting. For further information on Nature Portfolio policies, see our Editorial Policies and the Editorial Policy Checklist.

Statistics

For all statistical analyses, confirm that the following items are present in the figure legend, table legend, main text, or Methods section.

n/a	Confirmed	
☐	☒	The exact sample size (*n*) for each experimental group/condition, given as a discrete number and unit of measurement
☐	☒	A statement on whether measurements were taken from distinct samples or whether the same sample was measured repeatedly
☐	☒	The statistical test(s) used AND whether they are one- or two-sided<br>*Only common tests should be described solely by name; describe more complex techniques in the Methods section.*
☐	☒	A description of all covariates tested
☐	☒	A description of any assumptions or corrections, such as tests of normality and adjustment for multiple comparisons
☐	☒	A full description of the statistical parameters including central tendency (e.g. means) or other basic estimates (e.g. regression coefficient) AND variation (e.g. standard deviation) or associated estimates of uncertainty (e.g. confidence intervals)
☐	☒	For null hypothesis testing, the test statistic (e.g. F, t, r) with confidence intervals, effect sizes, degrees of freedom and P value noted<br>*Give P values as exact values whenever suitable.*
☒	☐	For Bayesian analysis, information on the choice of priors and Markov chain Monte Carlo settings
☒	☐	For hierarchical and complex designs, identification of the appropriate level for tests and full reporting of outcomes
☒	☐	Estimates of effect sizes (e.g. Cohen's *d*, Pearson's *r*), indicating how they were calculated

Our web collection on statistics for biologists contains articles on many of the points above.

Software and code

Policy information about availability of computer code

Data collection	Crystallography data was remotely collected from the Southeast Regional Collaborative Access Team (SER-CAT) using NoMachine and the program SERGUI at the Advanced Photon Source (APS), 22ID beamline (Argonne National Laboratories, Chicago, IL).<br>Luciferase reporter, BRET, and nanoPCA data was collected on a BioTek (BioTek, Winooski, VT) NEO using the GEN 5 v3.03 software.<br>qPCR experiments were collected using the StepOnePlus (Applied Biosystems, Foster City, CA, USA) and StepOne Software v2.3 .<br>TRIC experiments were collected and analyzed using the Dianthus NT.23 Pico (NanoTemper Technologies, München, Germany) instrument and DI.Control v1.0.2 and DI.Screening Analysis v1.0.1..
Data analysis	Crystallography data was processed and scaled using HKL-2000 and phased by molecular replacement using Phaser-MR from PHENIX v1.17.1 using a previously published PC-TP structure as a reference model. Models were built using COOT v 0.8.9.2, and refined using PHENIX v 1.17.1 and PDB_REDO. Structures were visualized using PyMOL v 2.1.1 (Schrödinger, LLC).<br>Statistical analysis was performed using GraphPad Prism 8.0.<br>Untargeted lipidomics analysis was performed using lipid search V4.2

For manuscripts utilizing custom algorithms or software that are central to the research but not yet described in published literature, software must be made available to editors and reviewers. We strongly encourage code deposition in a community repository (e.g. GitHub). See the Nature Portfolio guidelines for submitting code & software for further information.

Data

Policy information about availability of data

All manuscripts must include a data availability statement. This statement should provide the following information, where applicable:

- Accession codes, unique identifiers, or web links for publicly available datasets
- A description of any restrictions on data availability
- For clinical datasets or third party data, please ensure that the statement adheres to our policy

Data available in a publicly accessible repository that does not issue DOIs.
Publicly available datasets were analyzed in this study. This data can be found here: [will submit at acceptance]. Structure coordinates, and diffraction data have been deposited in the PDB under accession codes PDB ID [7U9D]
PDB 1LN1 was utlized as a starting model for experimental phasing.
RNAseq was generated from this work and is deposited to Geo under the accession number: GSE224877 .

Human research participants

Policy information about studies involving human research participants and Sex and Gender in Research.

Reporting on sex and gender	N/a
Population characteristics	N/a
Recruitment	N/a
Ethics oversight	N/a

Note that full information on the approval of the study protocol must also be provided in the manuscript.

Field-specific reporting

Please select the one below that is the best fit for your research. If you are not sure, read the appropriate sections before making your selection.

☒ Life sciences ☐ Behavioural & social sciences ☐ Ecological, evolutionary & environmental sciences

For a reference copy of the document with all sections, see nature.com/documents/nr-reporting-summary-flat.pdf

Life sciences study design

All studies must disclose on these points even when the disclosure is negative.

Sample size	No Sample size calculation was performed a priori. Sample size for previous RNAseq experiments was set to be 3. Follow up characterization of mouse phenotypes for the newly generated L-Pctp-/- was performed with minimum of 4 mice per experiment and repeated this at least for times, the number of mice was variable according to pups availability and could range from a minimum of n=4 per group to n=7/8. This number would vary as the retroocular injection was difficult to initially perform. All cell experiments were performed with minimum of 3 biologically independent replicates. Previous work performed in our lab has shown sufficient power for these in cell and in vitro experiments using the previously described numbers.
Data exclusions	no data was excluded
Replication	All data present is the result of biologically independent replicates or stringent statistical measures (S/B & z' for HTS). All in vitro work was performed a minimum of 3 technical replicates and 3 biologically independent replicates. In vivo work was was performed with minimum of 4 mice per experiment and repeated this at least for times, the number of mice was variable according to pups availability and could range from a minimum of n=4 per group to n=7/8 .
Randomization	For quantification of lipid droplets, images were acquired in the Cohen lab and analyzed in the Ortlund lab. Analysis was performed using Fiji. Samples were randomized and later unblinded. Allocation of samples, organs collected and functional assays (such as PTT, ITT, blood used for biochemical assays etc) was random across the different independently performed experiment.
Blinding	All assessments were performed in a unblinded manner as set up and analysis were performed by the same individual.

Reporting for specific materials, systems and methods

We require information from authors about some types of materials, experimental systems and methods used in many studies. Here, indicate whether each material, system or method listed is relevant to your study. If you are not sure if a list item applies to your research, read the appropriate section before selecting a response.

Materials & experimental systems

n/a	Involved in the study
☐	☒ Antibodies
☐	☒ Eukaryotic cell lines
☐	☐ Palaeontology and archaeology
☐	☒ Animals and other organisms
☐	☐ Clinical data
☐	☐ Dual use research of concern

Methods

n/a	Involved in the study
☐	☐ ChIP-seq
☐	☐ Flow cytometry
☐	☐ MRI-based neuroimaging

Antibodies

Antibodies used	Polyclonal antibodies to PC-TP and Them2 were prepared as previously described (dilution, 1:1000) (1, 2). GAPDH antibody was from Novus Biologicals, Inc. (Catalog # NB100-56875) (dilution, 1:1000). Polyclonal goat anti-rabbit immunoglobulins from Agilent Dako (Catalog # P0448) (dilution, 1:5000) was applied as secondary antibody.
Validation	Antibodies used for this analysis have been previously validated:<br>1. Kanno K, Wu MK, Agate DS, Fanelli BJ, Wagle N, Scapa EF, Ukomadu C, et al. Interacting proteins dictate function of the minimal START domain phosphatidylcholine transfer protein/StarD2. J Biol Chem 2007;282:30728-30736.<br>2. Shoda J, Oda K, Suzuki H, Sugiyama Y, Ito K, Cohen DE, Feng L, et al. Etiologic significance of defects in cholesterol, phospholipid, and bile acid metabolism in the liver of patients with intrahepatic calculi. Hepatology 2001;33:1194-1205

Eukaryotic cell lines

Policy information about cell lines and Sex and Gender in Research

Cell line source(s)	Cell Lines were purchased from ATCC<br>HUH7: A well differentiated hepatocyte-derived cell line, originally taken from a male in 1980s<br>HEK293T: An immortalized female human embryonic kidney cells from the 1970s
Authentication	All cell lines were stored at -196 degree Celsius . Passage 2 was cultured till passage 30 and not used after. Samples were purchased and verified by ATCC prior to freezing initial aliquots, no further assessment was performed.
Mycoplasma contamination	Cell lines were not tested for microplasma contamination.
Commonly misidentified lines (See ICLAC register)	No commonly misidentified cell lines were used.

Palaeontology and Archaeology

Specimen provenance	N/a
Specimen deposition	N/a
Dating methods	N/a

☐ Tick this box to confirm that the raw and calibrated dates are available in the paper or in Supplementary Information.

Ethics oversight	N/a

Note that full information on the approval of the study protocol must also be provided in the manuscript.

Animals and other research organisms

Policy information about studies involving animals; ARRIVE guidelines recommended for reporting animal research, and Sex and Gender in Research

Laboratory animals	Tissue specific knockdown mice were created (Institute of Model Animal, School of Medicine, Wuhan University, Wuhan, China; Dr.

Laboratory animals	Hongliang Li, Director) using a LoxP/Cre system C56BL6 background mice with Pctp flanked by two LoxP sites (Pctpflox/flox) using a CRISPR/Cas9 set up. Pctp flox/flox were transduced with AAV8 harboring a vector for Cre recombinase driven by the TBG promoter to generate hepatocyte specific deletion of Pctp-/- (L-Pctp-/-). Similarly, control mice were treated with equivalent titter of empty AAV8. Mice were housed in a barrier facility on a 12 h light/dark cycle at ambient temperatures with free access to water and respective diet.
Wild animals	No wild animals were used in this study
Reporting on sex	All animals used were male mice.
Field-collected samples	No field collected samples were used in the study
Ethics oversight	Animal use and euthanasia protocols were approved by the Institutional Animal Care and Use Committee of Weill Cornell Medical College.

Note that full information on the approval of the study protocol must also be provided in the manuscript.

Clinical data

Policy information about clinical studies

All manuscripts should comply with the ICMJE guidelines for publication of clinical research and a completed CONSORT checklist must be included with all submissions.

Clinical trial registration	N/a
Study protocol	N/a
Data collection	N/a
Outcomes	N/a

Dual use research of concern

Policy information about dual use research of concern

Hazards

Could the accidental, deliberate or reckless misuse of agents or technologies generated in the work, or the application of information presented in the manuscript, pose a threat to:

No	Yes	
☒	☐	Public health
☒	☐	National security
☒	☐	Crops and/or livestock
☒	☐	Ecosystems
☒	☐	Any other significant area

Experiments of concern

Does the work involve any of these experiments of concern:

No	Yes	
☒	☐	Demonstrate how to render a vaccine ineffective
☒	☐	Confer resistance to therapeutically useful antibiotics or antiviral agents
☒	☐	Enhance the virulence of a pathogen or render a nonpathogen virulent
☒	☐	Increase transmissibility of a pathogen
☒	☐	Alter the host range of a pathogen
☒	☐	Enable evasion of diagnostic/detection modalities
☒	☐	Enable the weaponization of a biological agent or toxin
☒	☐	Any other potentially harmful combination of experiments and agents

ChIP-seq

Data deposition

- [ ] Confirm that both raw and final processed data have been deposited in a public database such as GEO.
- [ ] Confirm that you have deposited or provided access to graph files (e.g. BED files) for the called peaks.

Data access links<br>*May remain private before publication.*	N/a
Files in database submission	N/a
Genome browser session (e.g. UCSC)	N/a

Methodology

Replicates	N/a
Sequencing depth	N/a
Antibodies	N/a
Peak calling parameters	N/a
Data quality	N/a
Software	N/a

Flow Cytometry

Plots

Confirm that:

- [ ] The axis labels state the marker and fluorochrome used (e.g. CD4-FITC).
- [ ] The axis scales are clearly visible. Include numbers along axes only for bottom left plot of group (a 'group' is an analysis of identical markers).
- [ ] All plots are contour plots with outliers or pseudocolor plots.
- [ ] A numerical value for number of cells or percentage (with statistics) is provided.

Methodology

Sample preparation	N/a
Instrument	N/a
Software	N/a
Cell population abundance	N/a
Gating strategy	N/a

- [ ] Tick this box to confirm that a figure exemplifying the gating strategy is provided in the Supplementary Information.

Magnetic resonance imaging

Experimental design

Design type	N/a
Design specifications	N/a
Behavioral performance measures	N/a

Acquisition

Imaging type(s)	N/a
Field strength	N/a
Sequence & imaging parameters	N/a
Area of acquisition	N/a

Diffusion MRI ☐ Used ☒ Not used

Preprocessing

Preprocessing software	N/a
Normalization	N/a
Normalization template	N/a
Noise and artifact removal	N/a
Volume censoring	N/a

Statistical modeling & inference

Model type and settings	N/a
Effect(s) tested	N/a

Specify type of analysis: ☐ Whole brain ☐ ROI-based ☐ Both

Statistic type for inference (See Eklund et al. 2016)	N/a
Correction	N/a

Models & analysis

n/a	Involved in the study
☒	☐ Functional and/or effective connectivity
☒	☐ Graph analysis
☒	☐ Multivariate modeling or predictive analysis
